# Improving Protocol Design Feasibility to Drive Drug Development Economics and Performance

**DOI:** 10.3390/ijerph110505069

**Published:** 2014-05-12

**Authors:** Kenneth Getz

**Affiliations:** Center for the Study of Drug Development, School of Medicine, Tufts University, 75 Kneeland Street, Suite 1100, Boston, MA 02111, USA; E-Mail: Kenneth.Getz@tufts.edu; Tel.: +1-617-636-3487; Fax: +1-617-636-2425

**Keywords:** protocol design complexity, protocol amendments, extraneous protocol procedures, direct drug development costs, indirect drug development costs, protocol feasibility

## Abstract

Protocol design complexity has increased substantially during the past decade and this in turn has adversely impacted drug development economics and performance. This article reviews the results of two major Tufts Center for the Study of Drug Development studies quantifying the direct cost of conducting less essential and unnecessary protocol procedures and of implementing amendments to protocol designs. Indirect costs including personnel time, work load and cycle time delays associated with complex protocol designs are also discussed. The author concludes with an overview of steps that research sponsors are taking to improve protocol design feasibility.

## 1. Introduction

Research and Development leaders widely agree that among the many factors that drive the high and rising cost of drug development, one of the most significant is protocol design [1]. As the blueprint articulating project strategy and directing project execution performed by both internal and external personnel, protocol design is uniquely positioned to fundamentally and directly impact—positively or negatively—drug development efficiency and economics. 

Protocol design has historically been the domain of clinical research scientists. Under a resource-rich R&D operating environment, the regulatory approval of safe and effective medical products was the primary test for assessing study design quality. In the current resource-constrained operating environment, a primary test of quality study design seeks to exclude any protocol that substantially wastes scarce resources, gathers extraneous data, and exposes study subjects to unnecessary risk. 

Making matters worse, in the current environment, a rising number of stakeholders influence protocol design practice and quality. Not only are scientists, regulatory agencies, health authorities, operating managers and key opinion leaders informing study design decision making, but also patients/patient organizations, investigative site personnel, health care providers, policymakers and payers are increasingly playing a role. 

Interviews conducted among drug development executives suggest a number of considerations and pressures that protocol design decision-making must accommodate: New scientific understanding about chronic disease mechanisms and how to measure their progression and economic impact requires collecting more clinical data. Crowded classes of experimental therapies and the ongoing movement to develop stratified medicines compel research sponsors to collect more genetic and biomarker data. 

Clinical scientists and statisticians often add procedures to gather more contextual data to aid in their interpretation of the findings and to guide development decisions. Clinical teams are now collecting biomarker data, genetic material, data on economic and therapeutic outcomes, and companion diagnostic data.

Clinical teams routinely add procedures guided by the belief that the marginal cost of doing so, relative to the entire clinical study budget, is small when the risk of not doing so is high [1,2]. Additional clinical data is collected as a precautionary measure in the event that a study fails to meet its primary and key secondary objectives. Data from these procedures may prove valuable in *post-hoc* analyses that reveal new and useful information about the progression of disease, its treatment and new directions for future development activity. Clinical teams add procedures for fear that they may neglect to collect data requested by regulatory agencies and health authorities, purchasers and payers. Failure to collect requested data elements could potentially delay regulatory submission, product launch and product adoption. And medical writers and protocol authors also often permit outdated and unnecessary procedures into new study designs because they are routinely included in legacy protocol authoring templates and operating policies. 

Collectively, these factors have all contributed to the dramatic increase in protocol design complexity during the past decade. Research conducted by the Tufts Center for the Study of Drug Development (Tufts CSDD, Boston, MA, USA) documents this alarming trend (see Table 1) and characterizes rising levels of scientific complexity (e.g., number of endpoints; number of procedures performed; number of study volunteer eligibility criteria) and operating complexity (e.g., number of countries where clinical trials are conducted; number of investigative sites activated and monitored; number of patients screened and enrolled). 

In 2012, to demonstrate safety and efficacy, a typical phase III protocol had 170 procedures on average performed on each study volunteer during the course of 11 visits across an average 230-day time span. Ten years ago, the typical phase III protocol had an average of 106 procedures, nine visits and an average 187-day time span. For the typical phase III protocol conducted in 2012, study volunteers came from an average of 34 countries and 196 research centers, up from 11 countries and 124 research centers ten years ago. And in 2012, to qualify to participate in a typical phase III protocol, each volunteer had to meet 50 eligibility criteria -- up from an average of 31 inclusion and exclusion criteria ten years ago [1]. 

**Table 1:** Comparing Scientific and Logistical Complexity of a Typical Phase III Protocol Across Two Time Periods.

Design Characteristics (All Values are Means)	2002	2012
Total number of endpoints	7	13
Total number of procedures	106	167
Total number of eligibility criteria	31	50
Total number of countries	11	34
Total number of investigative sites	124	196
Total number of patients randomized	729	597

In an effort to reign in rising drug development costs, a growing number of pharmaceutical and biotechnology companies are looking to identify protocol design practices that can be modified. This article reviews two major Tufts CSDD studies that have quantified the direct cost impact of protocol design practices. This article also summarizes growing knowledge of the impact of protocol design complexity on indirect costs. At the conclusion of the article, steps that drug developers are taking to simplify protocol design and lower overall study costs are discussed.

## 2. Direct Costs Associated with Protocol Design Practices

### 2.1. Protocol Procedures

In a study completed in 2012, Tufts CSDD demonstrated that the cost of adding multiple individual protocol procedures, in the aggregate, is substantial. Fifteen mid-sized and large pharmaceutical and biotechnology companies participated in the study. Each company provided data on their phase II and III protocols targeting diseases across multiple therapeutic areas and executed by investigative sites dispersed globally since 2009. To minimize unusual and atypical designs, pediatric, medical device, orphan drug and extension studies were excluded from the sampling frame. In all, 116 unique phase II and III protocols having at least one procedure tied to a primary endpoint were analyzed [2]. 

Participating companies classified each protocol procedure according to the objective and endpoint it supported as defined by the clinical study report (CSR) and the study’s specific statistical analysis plan (SAP) along the following lines:

1. “Core” procedures—supported primary and/or secondary study objectives or primary or key secondary and safety endpoints.
2. “Required” procedures—supported screening requirements and compliance-related activity including drug dispensing, informed consent form review, and study drug return.
3. “Standard” procedures—are commonly performed during initial and routine study participant visits including medical history, height and weight measurement, adverse event assessment, and concomitant medication review.
4. “Non-Core” procedures—supported supplemental secondary, tertiary and exploratory endpoints, and safety and efficacy procedures not associated with a study endpoint or objective.

Participating companies classified 25,103 individual phase II and III protocol procedures. Direct cost data for 16,607 procedures was analyzed using Medidata Solutions PICAS^®^ database. Overall, half of the total procedures per protocol were classified as “Core” to the study. Wide variability in the incidence of procedures supporting endpoint classifications was observed across therapeutic areas. Protocols targeting endocrine and central nervous system (CNS) disorders contained a higher relative average number of procedures supporting supplementary, tertiary, and exploratory procedures (e.g., “Non-Core”) endpoints. Oncology protocols had the highest relative proportion of procedures supporting “Core” endpoints and objectives, and the lowest relative proportion supporting “Non-Core” endpoints. 

The distribution of direct costs was similar to that of categorized procedures: Overall, 47.9% of the total study budget on average was spent on the direct costs to administer “Core” procedures in phase II and phase III protocols. The direct cost to administer “Required” (regulatory compliance) and “Standard” procedures for phase III protocols was 22.7% and 12.0% of the study budget respectively.

For phase III protocols alone, approximately half of the total direct cost (46.7%) was spent to administer “Core” procedures; 18.6% on average was spent to administer “Non-Core” procedures; 22.7% to administer procedures supporting screening requirements and regulatory compliance; and 12% supported “Standard” procedures (See Table 2).

**Table 2:** Distribution of Procedures and Direct Costs per Procedure by End Point Classification.

Endpoint Type	Phase II Procedures	Phase III Procedures	Phase II Procedure Costs	Phase III Procedure Costs
Core	54.4%	47.7%	55.2%	46.7%
Required	8.0%	10.0%	16.3%	22.7%
Standard	19.7%	17.6%	15.4%	12.0%
Non-Core	17.9%	24.7%	13.1%	18.6%
Total	100%	100%	100%	100%

One out of five (22.1%) phase II and III protocol procedures, on average, supported tertiary and exploratory objectives and endpoints. The proportion of procedures collecting non-core data in phase III studies alone was even higher (24.7%). Non-core procedures consumed on average 19% of total direct costs for each phase III study, and 13% of total direct costs for each phase II study [2]. 

The estimated total cost to the pharmaceutical industry each year to perform procedures supporting “Non-Core” objectives and endpoints for all FDA-regulated phase II and III protocols is an estimated $4–$6 billion USD. This estimate is very conservative as it excludes all indirect costs for personnel and infrastructure required to capture, monitor, clean, analyze, manage and store extraneous protocol data and it does not include any estimate for the unnecessary risk to which patients may be exposed.

### 2.2. Protocol Amendments

A second Tufts CSDD study showed that the incidence of protocol amendments rises with an increase in protocol design complexity. Amendments that are implemented after protocols have been approved are commonplace, but they are widely viewed as a nuisance, resulting in unplanned study delays and additional costs. Although clinical research professionals view protocol amendments as major problems, it is perhaps more accurate to view amendments as attempts to address underlying protocol design problems and external factors impacting design strategies. 

Conducted in 2010, Tufts CSDD convened a group of seventeen mid-sized and large pharmaceutical and biotechnology companies each of whom provided protocol amendment data from 3,410 protocols approved between January 2006 and December 2008. The protocols were representative of multiple therapeutic areas. Protocols approved within the most recent 12-months were excluded from the study because these had not had enough time to accumulate amendments [3]. 

Amendments were defined as any change to a protocol requiring internal approval followed by approval from the Institutional Review Board (IRB), Ethical Review Board (ERB) or regulatory authority. Detailed data on 3,596 amendments containing 19,345 total protocol modifications was analyzed. Companies participating in this study assigned specific changes made per amendment yielding a total of 6,855 changes classified. Companies also provided the top causes for amendments and rated each amendment in terms of whether it was “Completely Avoidable”, “Somewhat Avoidable”, “Somewhat Unavoidable” or “Completely Unavoidable”.

Nearly all protocols required at least one amendment. Completed protocols across all phases had an average of 2.3 amendments, though later-stage phase II and III protocols averaged 2.7 and 3.5 amendments respectively. Each amendment required an average of 6.9 changes to the protocol. Therapeutic areas that had the highest incidence of amendments and changes per amendment included cardiovascular and GI protocols (See Table 3).

**Table 3:** Mean Number of Amendments and Changes per Amendment per Protocol by Phase.

Research Phase	Number of Amendments	Protocol Changes per Amendment
Phase I	1.9	5.6
Phase II	2.7	6.8
Phase III	3.5	8.5
Phase IIIb/IV	2.6	8.3
All phases	2.3	6.9

Of the 6,855 changes categorized, 16% were modifications made to the description and eligibility criteria of the patient population under investigation; 12% were adjustments made in the number and types of safety assessment procedures; 10% were edits and revisions made to the general information contained in the protocol (e.g., protocol title and study staff contact information). 

**Figure 1:**
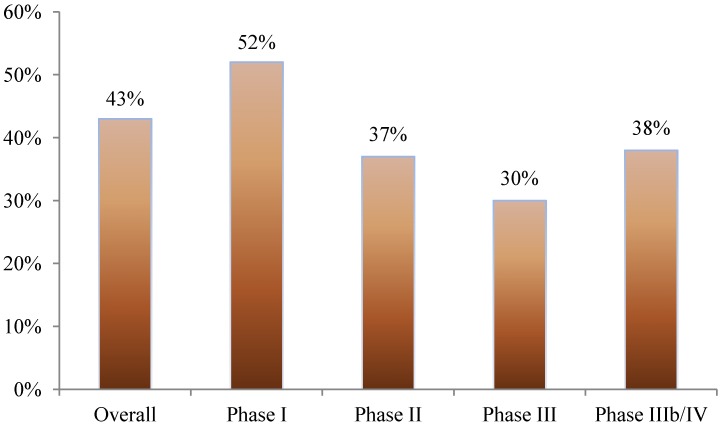
Proportion of protocol amendments occurring before the patient has received the first dose.

Nearly 40% of all amendments occurred before the first study volunteer received his or her first dose in the clinical trial. This was most pronounced in phase I studies where 52% of amendments occurred prior to beginning patient enrollment. In phase II, III, and IIIb/IV studies, 37%, 30%, and 38% of amendments occurred before first patient first dose, respectively (see Figure 1).

The most common cause of amendments was the availability of new safety information (20%), followed by requests from regulatory agencies to amend the study (19%) and changes in the study strategy (18%). Protocol design flaws and difficulties recruiting study volunteers were also top cited causes at 11% and 9% of categorized amendments, respectively. 

Two-thirds (63%) of amendments had causes that sponsor companies considered unavoidable, including amendments that were the result of new safety information, new regulatory requests, changes in the standard of care or study objectives. Nearly one-out-of-four protocol amendments (37%) was considered partially or completely avoidable. 

Based on data provided by participating companies, on average the direct cost to implement (*i.e.*, first patient resubmitted under the revised protocol) a single protocol amendment is approximately $500,000 (USD) in unplanned expense and adds 61 days to the project timeline. This figure undercounts the full economic impact as it does not include the cost of internal staff time dedicated to implementing each amendment, costs or fees associated with protocol language translation, and costs associated with resubmission to the local authority. 

Tufts CSDD estimates that the total cost for sponsors to implement “avoidable” protocol amendments in 2014 was approximately $2 billion (USD). This estimate is based on the incidence of amendments by phase for all active global FDA regulated clinical trials as reported by the agency; the direct cost to implement each amendment; and the proportion of amendments that is avoidable. This estimate does not include the billions of dollars realized annually in aggregate time-savings and earlier commercialization due to the elimination of each avoidable amendment [3].

To date, Tufts CSDD research on the direct cost impact of protocol design complexity has focused on non-core procedures. Tufts CSDD is currently conducting research looking at the direct cost of Core, Required and Standard procedures.

### 2.3. Indirect Costs

Tufts CSDD research is only beginning to quantify the indirect costs associated with protocol design complexity. These costs are no doubt many magnitudes higher than their estimated direct costs and include increasing cycle time and delays; full- and part-time staffing costs associated with project management and oversight, protocol administration and the gathering, monitoring, collecting, cleaning, analyzing, maintaining and storing of clinical data. 

### 2.4. Cycle Time

Research published in peer-reviewed and trade literature shows a very clear relationship between study design complexity and performance: Study designs that include a relatively large number of eligibility requirements and unique procedures conducted frequently have lower study volunteer recruitment and retention rates, take longer, and generate lower quality clinical data than designs without such features [4].

Tufts CSDD research has demonstrated that the average overall duration of clinical trials—from “Protocol Ready” to the last patient completing her last visit—is 74% longer for complex clinical trials. And whereas three-out-of-four volunteers are randomized following screening and two-thirds of volunteers complete simpler protocols; 59% are randomized and less than half (48%) complete complex protocols [4].

Other studies in the literature corroborate these findings and provide insights into causes of cycle time delays [5,6,7,8,9]. Clark found, for example, that the collection of excessive and unnecessary clinical data is driving longer study durations. The author warned that data collection and regulatory agency submission delays may ultimately harm regulatory approval rates [5]. 

Ross and colleagues conducted a comprehensive analysis of peer-reviewed academic studies and found that health professionals were less likely to refer, and patients less likely to participate in, more complex clinical trials [6]. Madsen showed that patients are significantly less likely to sign the informed consent form when facing a more demanding protocol design [7]. 

In a study conducted by Boericke and Gwinn, the higher the number of study eligibility criteria, the more frequent and longer were the delays in completing clinical studies [8]. Andersen and colleagues showed that volunteer drop-out rates are much higher among patients participating in more complex clinical trials. The authors cautioned that when volunteers terminate their participation early and are lost to follow-up, the validity of the study results may be compromised [9]. 

### 2.5. Investigative Site Work Burden

Rising protocol design complexity also places additional execution burden on study staff. Work burden to administer protocol procedures was assessed using an approach developed by Tufts CSDD in 2008 and adapted from Medicare’s Relative Value Unit (RVU) methodology [4]. The RVU scale was created by the Federal government in 1982 to determine reimbursement payment levels for physicians’ relative costs instead of prevailing charges for medical procedures. Tufts CSDD mapped medical procedure RVUs to clinical trial procedures to derive Work Effort Units (WEU) per protocol procedure. For procedures that were not already assigned a Medicare RVU, a panel of 10 physicians at the Tufts University School of Medicine was convened to estimate the time spent per protocol procedure. “Investigative Site Work Burden” is the product of WEUs per procedure and the frequency of the procedures that were conducted over the course of the protocol.

**Figure 2:**
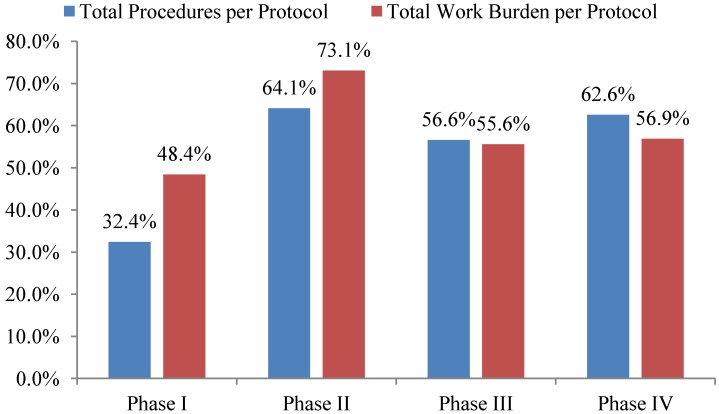
10-year overall growth (2002–2012) in total procedures and work burden per protocol by phase.

Total investigative site work burden to administer phase II protocol procedures grew the fastest—73%—during the ten year period 2002 to 2012 outpacing growth in the total number of procedures performed per protocol. The work effort required of site personnel to administer procedures supporting phase I, III and IV protocols increased 48%, 56% and 57% during the same ten year period respectively (see Figure 2). Protocols targeting diseases associated with immunomodulation, oncology, CNS, and cardiovascular required the highest work effort from investigative site personnel to administer. 

### 2.6. Study Monitoring, Data Management and Logistics Costs

Tufts CSDD studies have only just started to develop methodologies to measure the incremental indirect costs supporting study monitoring activity, data management and study logistics associated with complex protocol designs. In a preliminary analysis of regulatory submissions from nine major pharmaceutical companies, Tufts CSDD found that the average NDA in 1997 was nearly 70,000 pages in length and required 2 gigabytes of memory to store electronically. In 2012, the average NDA was almost 3.5 million pages and required 100 gigabytes of digital memory [10]. According to research from Medidata Solutions, a typical Phase III study collected nearly 1 million clinical data points in 2012, up from an estimated half that level 10 years ago [1]. 

Studies in the literature describe the impact of collecting too much data. Friedman and colleagues report that the high volume of data collected in today’s protocols distracts research scientists, compromises the data analysis process and ultimately harms data quality [11]. As more data is collected during protocol administration, error rates increase, according to Nahm *et al*. [12]. Barrett found that more procedures per protocol were associated with a higher incidence of unused data in NDA (New Drug Application) submissions [13]. Abrams *et al.* found that unused clinical data compromises the data analysis process [14]. 

Preliminary results from a 2014 Tufts CSDD study suggest that study monitors perform 100% source data verification on clinical data gathered from all non-core procedures and that one-third of all queries per case report form are associated with these less essential procedures. 

As mentioned earlier, Tufts CSDD research indicates that the number of countries and investigative sites where clinical trials are conducted has grown considerably. As clinical trials have become more globally dispersed, research sponsors have introduced numerous logistical complexities including delivering clinical supplies and collecting lab data from more remote locations; interacting with multiple regulatory agencies and health authorities; monitoring and overseeing sites in remote locations where infrastructure is more variable.

### 2.7. Steps to Optimize Study Design

During the past several years, a growing number of organizations have taken the initial steps of diagnosing their protocol design practices and comparing them with industry benchmarks. Sponsor organizations have referred to published data in peer-reviewed papers to compare internal study design practices with benchmarks. Commercially available software and consulting services are also available to assist sponsor companies in assessing the problem and implementing new practices to streamline and simplify study design [1].

In addition to diagnosing the problem, sponsor organizations are looking for ways to ensure that protocol feasibility is conducted more effectively. Whereas the scientific objectives solely dictated study design elements in the past, in an environment where resources and capital are far more limited, operating objectives now carry substantially more influence over design decisions. As such, the primary objective in optimizing protocol design is to perform great science that can be feasibly and cost-effectively executed.

Many sponsor organizations now solicit feedback from principal investigators, study coordinators and from patients to identify areas where study design feasibility can be improved prior to final approval of the protocol. Some of these feedback mechanisms are conducted as in-person meetings and focus groups while others are conducted online using social and digital media communities [15]. 

Protocol authoring practices received a valuable new reference resource in 2013. A group of international study design stakeholders issued a collection of checklists and guidelines designed to ensure that the purpose of protocol procedures are transparent and tied to core objectives and endpoints. The SPIRIT checklist was developed with input from 115 multi-disciplinary contributors from medical writing, journal editors, regulatory agencies, ethics committees, clinical research and health care professionals. 

The SPIRIT 2013 checklist and guidelines call for simple, minimalist designs that are clearly tied to core endpoints and objectives as defined by the clinical study report. Formatting conventions for various protocol elements (e.g., table of contents, glossary of terms, abbreviated terms) are also provided. The SPIRIT checklist has been pilot tested and can be downloaded for free [16]. 

A 2013 Tufts CSDD study documented the creation of internal governance committees charged with challenging clinical teams to improve protocol feasibility [1]. Pharmaceutical and biotechnology companies began establishing these committees 24 months ago in an effort to adopt a more systematic and long-term approach to optimizing study design. Committees are positioned within their respective organizations as objective governance and assessment mechanisms, offering guidance and input into the existing protocol review process without requiring organizations to alter legacy study design practices and procedures. 

The committees raise clinical team awareness of the impact that design decisions have on study budgets and on study execution feasibility. Committees typically provide input into the study design just prior to final protocol approval and they routinely offer insight into how protocol designs can be streamlined and better “fit to purpose”. Long term, internal facilitation committees may assist organizations in fundamentally improving their study design practices.

Nearly all of the facilitation committees are comprised of cross-functional representatives who volunteer their time. Committees representatives come from a variety of functions including clinical development; clinical operations; statistics; data management; medical writing; clinical pharmacology; regulatory; safety; and pharmacovigilance in addition to finance and/or procurement. Although some committees have more expansive goals than do others, all committees are charged with simplifying and streamlining protocols by reducing unnecessary procedures and the number of amendments. To meet these goals, committees assess the direct cost to perform non-core procedures and core procedures that may be conducted more frequently than necessary. 

Adaptive trial designs represent an optimization opportunity that has received limited attention from research sponsors to date. Adaptive trial designs are preplanned, typically through the use of trial simulations and scenario planning where one or more specified clinical trial design elements are modified and adjusted—while the trial is underway—based on an analysis of interim data. 

Tufts CSDD estimates that approximately one out of five (20%) late stage clinical trials are using a simple adaptive design approach. A much lower percentage—5%—is using more sophisticated adaptations (adaptive dose range studies and seamless phase II-III studies). Sponsor companies report that they expect the adoption of adaptive trial designs in earlier exploratory phase clinical trials to increase significantly over the next several years [17].

Study terminations due to safety and/or efficacy futility are the most common simple adaptive design used and they are more widely used at this time. Sponsor companies have found that early terminations due to futility are relatively easy to implement and a growing number of companies similarly view studies employing sample size re-estimation adaptations.

Although the concept of adaptive trial designs has been widely discussed for more than ten years, adoption has been slow for a variety of reasons. Internal organizational resistance appears to be the primary factor limiting more widespread adoption [17]. Based on interviews with company executives, regulatory agency receptivity to the use of adaptive trial designs does not appear to be as significant a barrier to adoption though agency clarity with regard to its position on the use of adaptive designs appears to be lacking. 

Clinical teams and operating functions perceive enrollment and logistical factors—specifically delays and disruptions in trial execution, patient participation and distribution of clinical supplies—as major barriers to adoption. Sponsors are also concerned about introducing bias following interim analyses; the lack of adaptive trial design experience among both internal development teams and external contract research organizations; gaps in infrastructure and technology to implement more sophisticated adaptive designs; and the limited capacity of independent data monitoring committees.

Adaptive trial designs hold promise in optimizing study design. Early study terminations due to futility and sample size re-estimation could save up to a hundred million dollars (USD) in direct and indirect costs annually per pharmaceutical company depending on clinical trial scope, when the trial is actually terminated, and on the sponsor’s overall implementation of this adaptive approach across the development portfolio. Perhaps the greatest impact from adaptive trial designs will come from improvements in late stage success rates. Even modest improvements in success rates for new molecular entities (NME) and new biologic entities (BME) represent billions of dollars in increased revenue potential for research sponsors. 

In the immediate term, adaptive trial designs are offering cross-functional teams direct insights into study design through scenario planning and trial simulation prior to finalizing the protocol. Rigorous upfront planning—similar to optimization practices for traditional study designs—is forcing organizations to challenge protocol feasibility prior to placing the protocol in the clinic.

## 3. Conclusions

Protocol design holds the key to fundamentally and sustainably transforming drug development performance, cost and success. Research conducted by Tufts CSDD and others contributes to our growing understanding of specific opportunities that can be implemented to optimize protocol design. There is no question that pharmaceutical and biotechnology companies will adopt and implement new approaches to test and modify study design feasibility with the goal of best executing excellent science. More flexible and adaptive trial designs are expected to play a growing role in helping to optimize study design by compelling sponsor companies to perform more rigorous upfront planning and simulation and to implement preplanned adaptions that may lower fixed operating costs and ultimately improve program success rates.
